# The huge burden of minor symptoms: the case of migraine headaches

**DOI:** 10.1590/S1516-31802005000600001

**Published:** 2005-11-01

**Authors:** Isabela M. Benseñor

Minor symptoms are a difficult part of our lives. They do not kill but they are responsible for poor quality of life and several disabilities. Headache is one of them. Among the types of headache, migraine is the champion in terms of days off work, with high prevalence among women.

Migraine can be a chronic symptom, with more than 15 days of pain each month, lasting for several months and years. Several studies in Brazil have evaluated the prevalence of migraine in different samples. Barea et al. observed that 9.9% of students aged 10-18 years had suffered migraine over the preceding year.^1^ Da Costa et al. and Sanvito et al. evaluated the prevalence of migraine among medical students and reported respectively 13% and 16%.^2,3^ Morillo et al. evaluated the prevalence of migraine in Latin America, finding an age-adjusted one-year female/male prevalence (%) of migraine of 6.1/3.8 in Argentina, 17.4/7.8 in Brazil, 13.8/4.8 in Colombia, 13.5/2.9 in Ecuador, 12.1/3.9 in Mexico and 12.2/4.7 in Venezuela.^4^

Although migraine is most common among women in their third and fourth decades of life, recent results from research among old people (65 or more years old) from low-income areas in São Paulo showed prevalence of 10%. Among these people with migraine, 20% reported that they had been suffering pain on more than 15 days per month for several years. (Benseñor, Scazufca and Menezes, unpublished data) Thus, the burden of migraine headaches is as high in Brazil as in other countries, and Bigal et al. have already reported on the high costs related to migraine borne by the Brazilian Health System.^5^

The diagnosing of migraine is done using specific criteria from the International Headache Society (IHS), as revised in 2003.^6^ Migraine headaches are predominantly unilateral, of moderate to severe intensity, with a pulsing/throbbing characteristic. They are worsened by routine physical activities, and are frequently associated with photophobia, phonophobia, nausea and vomiting. However, few doctors around the world have specific training for diagnosing migraine. A very interesting study published by Curtis et al. in the Archives of Internal Medicine in 2004 analyzed the clinical diagnosing of migraine based on the IHS criteria among 2,991 patients who sought medical care with a complaint of a history of self-described or physician-diagnosed “sinus” headache, without any previous diagnosis of migraine. Among these patients, 88% were diagnosed at the screening visit as fulfilling the IHS criteria for migraine (80% of the patients) or migrainous criteria (8% of the patients). The most common symptoms referable to the sinus area that were reported by the patients at the screening were sinus pressure (84%), sinus pain (82%), and nasal congestion (63%). Thus, migraine is frequently confounded with other symptoms.

Migraine is a very common disorder and most cases will be diagnosed and treated by general doctors. However, a Brazilian study that evaluated 414 patients with primary headaches (IHS groups 1, 2 and 3) showed that the correct diagnosis had previously been made among only 44.9% of the migraine patients, and most of them had undergone investigative procedures, especially electroencephalograms, that had been of no assistance in diagnosing their migraine. Preventive treatment was largely overlooked for migraine and other primary headaches.^7^

We can treat migraine in two manners: during an acute episode when the person is in pain, and prophylactically. For people with frequent attacks, or with a high burden of disabilities associated with migraine, prophylactic treatment is the solution. However, few doctors are trained to do this. At least three types of medication that can be used in migraine prophylaxis are readily available at primary care facilities: propranolol (a beta-blocker), tricyclic antidepressives (amitriptyline) and valproic acid. Propranolol is often used to treat hypertension, amitriptyline to treat depression and valproic acid to treat epilepsy, but all of them are good for the prophylactic treatment of migraine headaches.^8,9^

Prophylactic treatment for migraine can decrease the frequency and severity of migraine attacks. Medication is prescribed for daily use over a period of months to years. Some patients need medication for the rest of their lives, but with the important compensation of major improvement in their quality of life.

What we can do about this? In the general outpatient clinic of Hospital das Clínicas (Ambulatório Geral e Didático da Disciplina de Clínica Geral), within the School of Medicine, University of São Paulo, one hundred patients are interviewed from Monday to Friday each week, by students and residents of Internal Medicine. Of these one hundred patients, more than 10 have headaches and most of these are migraine headaches, of which 50% are chronic migraine. Since 1995, migraine headaches have been an issue that is discussed with all the students and residents that spend some time with us. Thus, training general doctors and medical students in migraine prophylaxis can be an important way of decreasing the burden of migraine headaches. Participation by university hospitals in the training of young doctors to treat primary headaches and especially migraine headaches is very important. Moreover, it is also very important to train primary care physicians in migraine prophylaxis. Simply by doing this, we can decrease people's burden of minor symptoms.

If a patient with migraine comes to a doctor's office complaining of pain, do not say, “It is just a migraine”: put this patient on prophylactic treatment. There is a yellow ipê tree in front of the emergency room of Hospital das Clínicas. Every August it flowers and brings hope to people. So, bring hope to a patient with migraine: prescribe prophylactic treatment.
